# Examining the referral of patients with elevated blood pressure to health resources in an under-resourced community in South Africa

**DOI:** 10.1186/s12889-023-17359-z

**Published:** 2024-02-08

**Authors:** Lia K. McNulty, Mark Stoutenberg, Andrea Kolkenbeck-Ruh, Amy Harrison, Thabiso Mmoledi, Daniel Katiyo, Mimi Mhlaba, Delisile Kubheka, Lisa J. Ware

**Affiliations:** 1 Department of Kinesiology, College of Public Health, Temple University, Philadelphia, PA USA; 2 Centre for Exercise Science and Sports Medicine, School of Therapeutic Sciences, Faculty of Health Sciences, University of the Witwatersrand, Wits Education Campus, Gauteng, South Africa; 3 Cardiovascular Pathophysiology and Genomics Research Unit, School of Physiology, Faculty of Health Sciences, University of the Witwatersrand, Johannesburg, South Africa; 4 School of Therapeutic Sciences, Faculty of Health Sciences, University of the Witwatersrand, Johannesburg, South Africa; 5 Phila Sonke Wellness Initiative, Dobsonville Stadium, Dobsonville, Johannesburg, South Africa; 6 SA MRC/Wits Developmental Pathways for Health Research Unit (DPHRU), School of Clinical Medicine, Faculty of Health Sciences, University of the Witwatersrand, Chris Hani Baragwanath Academic Hospital, Soweto, South Africa; 7 Wits Health Hubb, Wits Health Consortium, Johannesburg, South Africa; 8 Department of Sport and Exercise Sciences, Faculty of Social Sciences and Health, Durham University, Durham, UK

**Keywords:** Community health workers, Home visit, Hypertension, Elevated blood pressure, Non-communicable Diseases, Physical activity, Referral, South Africa

## Abstract

**Background:**

Low-and-middle income countries face a disproportionate burden of non-communicable diseases (NCDs) that threaten to overwhelm under-resourced health systems. Community health workers (CHWs) can promote NCD prevention, reach patients, and connect them to local community health resources; however, little has been done to examine how referrals to these resources are utilized by community members. The purpose of this study is to examine the use of referrals to community-based health resources and investigate the factors influencing patient utilization of referrals connecting them to appropriate health resources for elevated blood pressure (BP).

**Methods:**

CHWs conducted home visits, which included BP screening and brief counseling, with community members in Soweto, South Africa. Participants with elevated (systolic BP: 121–139/ diastolic BP: 81–89 mmHg) or high (≥ 140/90 mmHg) BP were referred to either a local, community-based physical activity (PA) program managed by a non-governmental organization or local health clinics. The number of participants that received and utilized their referrals was tracked. Follow-up interviews were conducted with individuals given a referral who: (1) went to the PA program, (2) did not go to the PA program, (3) went to a clinic, and (4) did not go to a clinic. Interviews were transcribed and analyzed to identify common themes and differences between groups regarding their decisions to utilize the referrals.

**Results:**

CHWs visited 1056 homes, with 1001 community members consenting to the screening; 29.2% (n = 292) of adults were classified as having optimal BP (≤120/80 mmHg), 35.8% (n = 359) had elevated BP, and 35.0% (n = 350) had high BP. One hundred and seventy-three participants accepted a referral to the PA program with 46 (26.6%) enrolling. Five themes emerged from the interviews: (1) prior knowledge and thoughts on BP, (2) psychosocial factors associated with BP control, (3) perception about receiving the referral, (4) contextual factors influencing referral utilization, and (5) perceived benefits of utilizing the referral.

**Conclusion:**

CHWs can successfully increase community members’ access to health resources by providing appropriate referrals. However, greater attention needs to address community members’ barriers and hesitancy to utilize health resources.

**Supplementary Information:**

The online version contains supplementary material available at 10.1186/s12889-023-17359-z.

## Introduction

Low- and middle-income countries (LMICs) are disproportionally burdened by noncommunicable diseases (NCDs) where 77% of all NCD-related deaths occur [1]. Beyond mortality, NCDs have a significant economic impact in LMICs, such as high medical costs that lead to increased out-of-pocket expenses and altered household spending patterns [2]. As many as 11% of individuals in LMICs would be impoverished if they had to purchase even the lowest-priced diabetes medication [3]. In South Africa, 85% of individuals do not have private health insurance, adding to the elevated medical costs [4]. In addition to individual level impacts, NCDs also effect health systems requiring a shift in healthcare budgets towards related treatment [5]. Health systems in LMICs are often overwhelmed with insufficient resources to address increasing NCD rates including a lack of trained professionals, insufficient budgets, decreased access to medications, plus the burden of other acute health issues [6, 7]. Hypertension, in particular, presents a tremendous population health challenge as only 8% of individuals in LMICs have controlled blood pressure (BP) [8]. Diagnosis and treatment of hypertension in South Africa remains low even though estimates of the prevalence of hypertension have grown to 40% of the population [4], and with even higher rates in some areas. A study assessing the profile of stroke survivors in low-income neighborhood in South Africa found that 69.8% of patients were hypertensive [9].

Proper care and treatment can help control NCDs and reduce the rate of NCD-associated mortality [10]. Across multiple guidelines, lifestyle changes, including increasing physical activity (PA), are typically the first recommendation for patients with BP above the optimal range of < 120/80 mmHg [11, 12]. Previous work has shown that engaging in exercise programs, as part of a lifestyle change, significantly reduces BP in individuals from LMICs [13]. However, in many South African low-income communities, it may not be viewed as culturally appropriate or safe to exercise outside and facilities designed to promote safe exercise are often not available, not known about, or vandalized [14]. Guidelines further recommend that patients at higher cardiovascular risk (those with elevated BP, additional cardiovascular risk factors, or diabetes) should receive pharmacological treatment for hypertension, alongside the lifestyle changes [11, 12]. Despite evidence in Sub-Saharan Africa of the potential effectiveness of medication use [15], insufficient access to healthcare resources remains a persistent barrier to treatment in LMICs. Additionally, individuals attempting to seek care can face multiple challenges accessing health resources including a lack of availability (e.g., limited government clinic hours, long wait times, lack of staff), affordability (e.g., high private clinic fees), and acceptability (e.g., perceptions and satisfaction of care) [16]. One potential solution to improve access to care and BP management in low-resource communities is to enhance the connection of patients to existing health resources for prevention and treatment efforts.

Community health workers (CHWs) can act as ‘connectors’ within their communities, linking and referring individuals to health services, providing health education and support, and helping patients manage and navigate local healthcare systems [17, 18]. Within LMICs, CHWs have traditionally provided support for maternal and child health, and infectious disease [17]. CHWs have also proven successful at increasing PA levels through health promotion efforts and, more recently, helped prevent and manage NCDs [19]. Furthermore, the location of CHWs within the community expands the reach of the health system beyond its traditional walls to improve access to BP treatment and prevention efforts in under-resourced communities [20]. CHW-led home visits have been shown to be acceptable and well-received for BP management by under-resourced communities in South Africa [21].

Despite the potential for increasing access to health services through CHW-led home visits, there is a lack of evidence on how community members with elevated BP would receive and utilize CHW referrals to local health resources. Therefore, this study assessed community members’ acceptance and utilization of referrals provided to them by CHWs during a home visit in Soweto, South Africa. Additionally, our second aim was to understand the behavioral and contextual factors that influenced community member decisions to utilize the referrals.

## Methods

As part of this mixed methods study, CHWs conducted home visits to community members in Soweto, South Africa between May and June of 2022. During the home visits, CHWs followed a standardized protocol for BP measurement, provided community members with relevant, brief guidance based on the results of the screening, and a referred community members to appropriate health resources to help manage their BP. Follow-up interviews were used to discuss participants’ decision to utilize (or not) the referral that they received.

### Study location, participants, and community health workers

The home visits were conducted with community members of the Dobsonville township located in Soweto, South Africa near the facility that hosted the community-based PA programme. The facility is in an area marked by historical deprivation and crime with 165 reported assaults, 50 sexual offences, 120 contacted related crimes occurring from April to June of 2021 [22].

The trainee CHWs involved in this study were young adults (age 18–30 years), undergoing professional training in health promotion, health behavior change, and community health screening. This training was a part of an accredited CHW qualification under the South African National Qualification Framework Health Promotion Office/Community Health Worker [23]. Standard minimum entry requirements for community health work were applied to all CHW trainings. The training program was a part of a youth employment initiative/research program operated by the Wits Health Hubb (witshealthhubb.org). Individuals were eligible for this program if they were from the local community and not in employment, education, or training (NEET) [24], and selected based on the results of basic competency tests that assessed language and mathematics and interviews that assessed core competencies necessary for the intended role.

### Determining study eligibility

The trainee CHWs went door-to-door in pairs to conduct the home visits, following a standardized screening protocol. Once the community member answered the door, the CHWs explained the reason for their visit, requested permission to conduct a COVID screening, and assessed their eligibility for the study. Community member eligibility criteria to participate in the screening included: (1) being at least 18 years old; (2) willingness to provide their informed consent; and (3) not displaying any symptoms of COVID-19 (determined by an infrared, non-contact thermometer reading of ≤ 37.5 °C or the presence of symptoms). If participants did not display COVID symptoms, had a normal temperature, and met the eligibility criteria, CHWs then entered the home and proceeded to follow a standardized BP screening and counseling process. The informed consent process throughout this study consisted of three parts: (1) participants provided their electronic signature for the home screening, (2) participants were asked for their consent to be recontacted at the end of the home visit, and (3) participants were asked for their consent before follow-up interviews began. Throughout, the research process is explained to community members in a language that they are most comfortable with as the CHWs in this study are all fluent in at least five languages.

### Measurement protocol and referral process

Blood pressure measurements during the home visits were conducted following the May Measurement Month (MMM) 2022 protocol [25] and the CHWs are trained to take BP following the International Society of Hypertension Global Hypertension Practice Guidelines [11]. To begin, sociodemographic data (age, self-reported ethnicity, education level, sex), self-reported medical history and medication, and tobacco and alcohol use data was collected. Next, physical activity levels were assessed using the Physical Activity Vital Signs (PAVS) Questionnaire [26]. Then, seated, left arm brachial BP was measured three times using an Omron automated device (M6; Omron Healthcare, Kyoto, Japan) following an initial five minutes of rest and one minute between each measure. The first reading was discarded and the second and third readings were averaged to obtain the BP result that was provided to the participant. Finally, brief counseling on health lifestyles (e.g., healthy diets and regular physical activity) and the provision of referrals were based on the average BP readings obtained, not self-reported medication use or prior medical history data. All participants were provided with MMM BP education resources with a written copy of their BP reading.

Participants with a systolic BP (SBP) of < 120 mmHg and a diastolic BP (DBP) of < 80 mmHg (categorized as optimal BP) were advised to follow the guidance in the MMM education resources. Participants with an elevated BP (SBP of 121–139 or DBP of 81–89 mmHg), were offered a referral to a local, medically-supervised, community-based PA program managed by the non-governmental organization Phila Sonke. Participants with high BP (≥140/90 mmHg), were offered a referral to attend a local health clinic or the medically-supervised PA program. At the end of the visit, CHWs asked the individuals if they would be willing to participate in a follow-up in-person interview. A member of the research team attempted to provide a reminder phone call to individuals who accepted the referral within 10 days of the home visit, encouraging them to attend the health service to which they were referred.

Participants were given up to 12 weeks after the home visit to utilize their referral. Enrollment registration in the PA program was tracked through the paper referrals provided at program registration. Utilization of clinic referrals was assessed by self-report during the follow-up telephone calls used to schedule interviews with participants who were able to be contacted. Study data were collected and managed using REDCap electronic data capture tools hosted at the University of the Witwatersrand [27].

### Individual interviews

Consenting participants that received a referral were contacted by phone and invited to participate in an in-person individual interview approximately 7–12 weeks after receiving the initial referral. Three separate attempts were made to contact participants by phone. Attempts were made to conduct interviews with participants from each of four categories: (1) those who were referred and went to the PA program; (2) those who were referred but did not go to the PA program; (3) those who were referred and went to a local health clinic; and (4) those who were referred but did not go to a local health clinic. A semi-structured interview guide was developed based on Andersen’s Expanded Behavioural Model of Health Service Use [28] with minor adaptations made for each participant category (Additional file 1). The model is composed of three main factors and associated sub-factors: (1) psychosocial factors (attitudes, knowledge, social norms, and perceived control), (2) enabling factors (availability of support and financial resources), and (3) need (perceived severity of health condition). The interview guide consisted of 30 questions and lasted a maximum or 30 min. Each interview was audio recorded, transcribed, and translated verbatim with all personal identifiers removed.

### Data analysis

The number of households visited in the area was documented in REDCap by the CHWs conducting the home visits. The CHWs tracked: the number of homes visited, community members that gave consent to participate in the study, and the number and type of referrals given. The statistical data analysis was conducted in SPSS version 28.0.1.0. For the continuous variables that were collected (i.e., age, years of education, SBP and DBP), visual inspection of histograms informed the normality of data, and the mean and standard deviation were reported. Median and interquartile ranges were reported for non-normally distributed data, while absolute numbers and percentages were reported for categorical variables. A multivariable logistic regression analysis was conducted to determine factors predicting PA referral utilization. To analyze the individual interviews, deductive coding was used throughout, and an initial codebook was developed from a subset of transcripts. Two research team members coded the first two transcripts and refined the codebook as more transcripts were reviewed. Five interviews were then independently coded by the two research team members. The initial rate of agreement was 81.2%; discrepancies were discussed until consensus was achieved. All interview transcripts were then uploaded to Dedoose [SocioCultural Research Consultants (SCRC), London version 7.0.23] to support qualitative analysis and the transcript coding was split between the two coders. The study team reviewed the coded data together multiple times to identify meaningful themes and differences between categories of participants and by sex.

## Results

### Study sample and characteristics

**Fig. 1:**
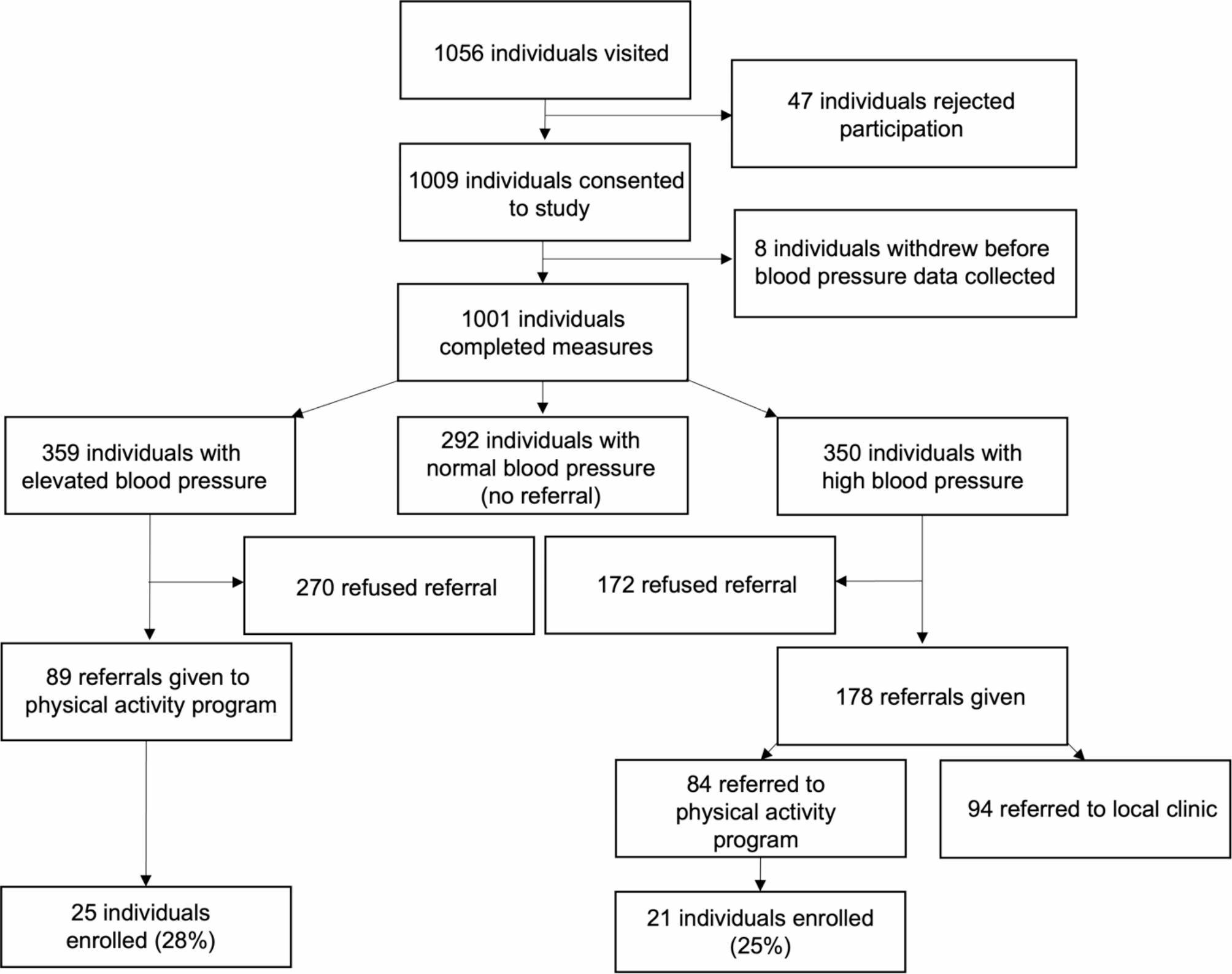
Home visit and referral flow diagram

CHWs visited 1056 homes in Soweto with 1001 eligible community members consenting to participate and complete the measurements during the home visit (Fig. 1). Of the 1001 participants, 59.3% were female with a median age of 46.5 years for females and 43.0 years for males. Nearly all participants (99.8%) self-identified as Black and more than half (56.8% overall; 55.5% of males; 57.7% of females) reported completing 7–12 years of education. Of the 1001 participants, 29.2% had optimal BP (n = 292; 61.3% female), 35.8% had elevated BP (n = 359; 56.5% female) and 35.0% had high BP (n = 350; 60.6% female) (Table 1).

**Table 1:** Distribution and characteristics of the study population

Characteristic	Total Study Sample (n = 1001)		Optimal BP (≤ 120/80 mmHg) (n = 292)		Elevated BP (121–139 or 80–89 mmHg) (n = 359)		High BP (BP ≥ 140/90) (n = 350)	
	Males (n = 407)	Females (n = 594)	Males (n = 113)	Females (n = 179)	Males (n = 156)	Females (n = 203)	Males (n = 138)	Females (n = 212)
**Socio-demographic**								
Age, years	43.0 (31.0–56.0)	46.5 (32.0–62.0)	39.0 (27.0–49.0)	36.0 (24.0–49.0)	40.0 (29.3–49.8)	48.0 (34.0–61.0)	53.0 (40.8–62.3)	54.5 (40.0-65.8)
Self-diagnosed ethnicity, n (%)								
Black African	405 (99.5)	594 (100)	113 (100)	179 (100)	155 (99.4)	203 (100)	137 (99.3)	212 (100)
White	1 (0.2)	-	-	-	1 (0.6)	-	-	-
Mixed race	1 (0.2)	-	-	-	-	-	1 (0.7)	-
Number years education, n (%)								
No education,	16 (3.9)	26 (4.4)	1 (0.9)	6 (3.4)	10 (6.4)	8 (3.9)	5 (3.6)	12 (5.7)
1–6 years,	53 (13.0)	81 (13.6)	14 (12.4)	26 (14.5)	16 (10.3)	18 (8.9)	23 (16.7)	37 (17.5)
7–12 years,	226 (55.5)	343 (57.7)	64 (56.6)	98 (54.7)	90 (57.7)	125 (61.6)	72 (52.2)	120 (56.6)
> 12 years,	107 (26.3)	138 (23.2)	34 (30.1)	46 (25.7)	38 (24.4)	50 (24.6)	35 (25.4)	42 (19.8)
Missing data	5 (1.2)	6 (1.0)	-	3 (1.7)	2 (1.3)	2 (1.0)	3 (2.2)	1 (0.5)
**Anthropometry**								
Height, cm	168.0 (162.2-174.5)	158.0 (152.0-163.0)	167.0 (162.0-174.8)	158.0 (152.0-163.0)	168.5 (164.0-174.9)	158.0 (152.0-163.0)	168.0 (162.0-174.0)	157.6 (151.3-162.1)
WC, cm	80 (71–89)	88 (77–103)	76 (70–85)	81 (72–89)	81 (72–88)	88 (76–103)	85 (72–95)	98 (85–110)
Central obesity (WHtR ≥ 0.5), n (%)	178 (60.1)	409 (86.5)	36 (44.4)	110 (76.9)	76 (64.4)	140 (86.4)	66 (68.0)	159 (94.6)
*Unable to collect, n (%)	111 (27.3)	121 (20.4)	32 (28.3)	36 (20.1)	38 (24.4)	41 (20.2)	41 (29.7)	44 (20.8)
**Blood pressure**								
SBP, mmHg	124 (115–138)	123 (112–136)	111 (105–116)	108 (103–114)	124 (120–129)	124 (117–129)	143 (136–157)	142 (131–159)
DBP, mmHg	83 (77–91)	83 (77–91)	74 (69–78)	74 (70–78)	83 (79–86)	83 (80–86)	95 (91–101)	95 (91–101)
Last BP Check, n (%)								
Never	150 (36.9)	143 (24.1)	51 (45.1)	63 (35.2)	58 (37.2)	43 (21.2)	41 (29.7)	37 (17.5)
Within last 12 months	164 (40.3)	333 (56.1)	45 (39.8)	87 (48.6)	59 (37.8)	115 (56.7)	60 (43.5)	131 (61.8)
Over 12 months	87 (21.4)	112 (18.9)	15 (13.3)	28 (15.6)	37 (23.7)	40 (19.7)	35 (25.4)	44 (20.8)
Missing data	6 (1.5)	6 (1.0)	2 (1.8)	1 (0.6)	2 (1.3)	5 (2.5)	2 (1.4)	-
**Self-reported medical history**								
Hypertension prior diagnosis, n (%)	53 (13.0)	184 (31.0)	6 (5.3)	22 (12.3)	13 (8.3)	67 (33.0)	34 (24.6)	95 (44.8)
On treatment, n (%)	50 (12.3)	172 (29.0)	6 (5.3)	22 (12.3)	11 (7.1)	65 (32.0)	33 (23.9)	85 (40.1)
Diabetes mellitus, n (%)	14 (3.4)	41 (6.9)	5 (4.4)	6 (3.4)	5 (3.2)	15 (7.4)	4 (2.9)	20 (9.4)
Previous heart attack, n (%)	3 (0.7)	4 (0.7)	2 (1.8)	1 (0.6)	1 (0.6)	1 (0.5)	-	2 (0.9)
Previous stroke, n (%)	8 (2.0)	15 (2.5)	4 (3.5)	3 (1.7)	1 (0.6)	4 (2.0)	3 (2.2)	8 (3.8)
**Behavioural health factors**								
Tobacco Use, n (%)								
Current use	220 (54.1)	95 (16.0)	64 (56.6)	25 (14.0)	82 (52.6)	39 (19.2)	74 (53.6)	31 (14.6)
Past use	72 (17.2)	123 (20.7)	21 (18.6)	36 (20.1)	32 (20.5)	41 (20.2)	19 (13.8)	46 (21.7)
Never used	113 (27.8)	373 (62.8)	28 (24.8)	116 (64.8)	41 (26.3)	122 (60.1)	44 (31.9)	135 (63.7)
Missing data	2 (0.5)	3 (0.5)	-	2 (1.1)	1 (0.6)	1 (0.5)	1 (0.7)	-
Alcohol consumption, n (%)								
Daily	35 (8.6)	9 (1.5)	9 (8.0)	2 (1.1)	12 (7.7)	4 (2.0)	14 (10.1)	3 (1.4)
1–6 times per week	93 (22.9)	55 (9.3)	27 (23.9)	21 (11.7)	34 (21.8)	13 (6.4)	32 (23.2)	21 (9.9)
1–3 times per month	156 (38.3)	175 (29.5)	39 (34.5)	52 (29.1)	66 (42.3)	61 (30.0)	51 (37.0)	62 (29.2)
Never/rarely	123 (30.2)	353 (59.4)	38 (33.6)	104 (58.1)	44 (28.2)	123 (60.6)	41 (29.7)	126 (59.4)
Missing data	-	2 (0.3)	-	-	-	2 (1.0)	-	-
PA < 150 min/week, n (%)	145 (35.6)	204 (34.3)	35 (31.0)	42 (23.5)	57 (36.5)	79 (38.9)	53 (38.4)	83 (39.2)

^*^Unable to collect anthropometry measurements as no private space available within the household

All data are presented as median and IQR (interquartile range) unless otherwise stated

BP: blood pressure; DBP: diastolic blood pressure; PA: physical activity; SBP: systolic blood pressure; WC: waist circumference; WHtR: waist to height ratio

Of the 359 participants identified with elevated BP, 89 were provided with referrals to the PA program with 25 (28.1%) ultimately enrolling. Of the 350 participants with high BP, 84 were provided with referrals to the PA program, with 21 (25.0%) ultimately enrolling. In total, 173 participants (52.6% female) were referred to the PA program with 26.5% (n = 46; 43.5% female) ultimately enrolling (Table 2). Another 94 participants with high BP were referred to a local health clinic; 69 of whom (73.4%) could not be contacted to determine referral utilization. Of the 24 individuals that were contacted, 25.0% (n = 6; 2 females) reported using the referral and attending a local health clinic. Furthermore, a multivariable logistic regression analysis, adjusting for age, revealed a significant association between education level and PA referral utilization, where participants with more than 12 years of education were significantly more likely to utilize that referral compared to those with 12 years or less education (OR 0.60; 95% CI 0.39–0.92; p = 0.019). Additionally, males were significantly more likely to utilize the PA referral than females (OR 0.69; 95% CI 0.49–0.96; p = 0.030). A total of 442 individuals with elevated or high BP (44.2%) refused to take a referral to either the PA program or a local health clinic.

**Table 2:** Distribution and characteristics of participants that received and utilized referral to the physical activity program

Characteristic	Referred to PA program (n = 173)		Enrolled in PA program (n = 46)		Did not enroll in PA program (n = 127)	
	Males (n = 82)	Females (n = 91)	Males (n = 26)	Females (n = 20)	Males (n = 56)	Females (n = 71)
**Socio-demographics**						
Age, years (median, IQR)	38.0 (29.0–52.0)	49.0 (36.0–60.0)	37.5 (27.0–54.0)	39.0 (29.3–60.0)	38.0 (29.3–51.8)	52.0 (38.0–60.0)
Education						
<12 years, n (%)	67 (81.7)	75 (82.4)	18 (69.2)	15 (75.0)	49 (87.5)	(84.5)
≥12 years, n (%)	15 (18.3)	16 (17.6)	8 (30.8)	5 (25.0)	7 (12.5)	11 (15.5)
**Anthropometry**						
Waist circumference, cm	80.0 (71.0–89.0)	94.0 (80.0-107.8)	85.0 (74.5–98.0)	98.5 (89.0-110.8)	75.5 (70.0-86.3)	93.0 (80.0-107.8)
Central obesity (WHtR ≥0.5), n (%)	41 (50.0)	66 (72.5)	16 (61.5)	12 (60.0)	25 (44.6)	54 (76.1)
*Unable to collect, n (%)	15 (18.3)	20 (21.9%)	5 (19.2)	8 (40.0)	10 (17.9)	12 (16.9)
**Blood pressure**						
Last BP measure < 12 months, n (%)	65 (79.3)	74 (81.3)	19 (73.1)	19 (95.0)	46 (82.1)	55 (77.5)
Last BP measure ≥ 12 months, n (%)	17 (20.7)	17 (18.7)	7 (26.9)	1 (5.0)	10 (17.9)	16 (22.5)
SBP, mmHg (mean ± SD)	133 ± 7	133 ± 11	131 ± 5	132 ± 12	133 ± 9	133 ± 11
DBP, mmHg (mean ± SD)	86 ± 9	90 ± 9	86 ± 9	91 ± 9	85 ± 9	90 ± 9
Classification of BP reading, n (%)						
Optimal < 120/80)	-	-	-	-	-	-
Elevated (120–139/80–89)	48 (58.5)	41 (45.1)	15 (57.7)	10 (50.0)	33 (58.9)	31 (43.7)
High (≥ 140/90)	34 (41.5)	50 (54.9)	11 (42.3)	10 (50.0)	23 (41.1)	40 (56.3)
**Physical activity**						
PA < 150 min/week, n (%)	49 (59.8)	56 (61.5)	14 (53.8)	10 (50.0)	35 (62.5)	46 (64.8)
PA ≥ 150 min/week, n (%)	33 (40.2)	35 (38.5)	12 (46.2)	10 (50.0)	21 (37.5)	25 (35.2)

^*^Unable to collect anthropometry measurements as no private space available within the household

BP: blood pressure; DBP: diastolic blood pressure; IQR: interquartile range; PA: physical activity; SBP: systolic blood pressure; SD: standard deviation

### Individual interviews

Of the 267 participants who received referrals, 55.4% (n = 148; 57% female) were willing to receive a follow-up call, of which 56.8% (n = 84; 58% female) were willing to be interviewed about their experience. The research team was successful in contacting 68 individuals, conducting a total of 35 interviews with 9 participants who were referred and went to the PA program; 14 participants who were referred but did not go to the PA program; 5 participants who were referred and went to a local health clinic; and 7 participants who were referred but did not go to a local health clinic. Analysis of the interviews revealed five main themes influencing community members’ decision on whether to utilize their referral: (1) prior knowledge and thoughts on BP, (2) psychosocial factors associated with BP control, (3) perceptions about receiving the referral​​, (4) contextual factors influencing utilization of the referral​​, and (5) perceived benefits of utilizing the referral​​. Sample quotes representing these themes and a summary of the main themes are presented in Tables 3 and 4, respectively.

**Table 3:** Representative quotes of themes emerging from follow-up interviews

Theme	Sub-themes	Quotes
Prior knowledge and thoughts on BP	Knowledge about high BP	“I don’t know much, all I know is that it’s not a well-known disease for people around my age, it’s not common.” “Eh I don’t have much knowledge about that.” “I do not have full knowledge with regards to such things maybe because I have not been on that path or maybe I have not experienced such things.”
	Causes/symptoms	“I know that it kills, you get it by eating unhealthy food.” “Oh, you can get a headache, dizziness sometimes and sweating then you feel hot.” “I think that maybe it’s speaking out of anger too much, or maybe the food we eat.”
	How to lower high BP	“And for it to be better, you have to exercise, stop using salt, oil and spices, things that are difficult to stop doing.” “Keep on being fit, to train, exercise and to try and eat healthy food.” “I should continue walking, reduce oil in my foods, and reduce my alcohol intake, yeah!”
	Previous attempts to lower BP	“I didn’t do anything until you guys’ came.” “Yes, there is medicating I’m taking but I don’t take it frequently.”
Psychosocial factors associated with BP control	Social norms of high BP	“Yes, there are. There’s my neighbor, a lot of us at home have it. My brother from (mention’s name of city).” “But in the yard, there are there, the elderly.” “She started taking treatment”
	Seriousness of high BP	“No, it doesn’t give me stress because I haven’t experienced what I see from other people.” “It is quite serious considering the type of food I eat and the lifestyle that I live it is quite serious.” “As I said that it’s dangerous, I saw from my mother when she died.”
	Permission to use referral	“No. I can go by myself.” “I don’t need to ask for permission.”
Perceptions of receiving the referral	Thoughts about receiving referral	“No, that time I was excited that I’m trying out new things. I was very excited.” “it’s very, very helpful.” “I was frightened, I was frightened but they consoled me and said it’s high but it’s not bad.” “I felt like it was a step in the right direction in terms of health in general.”
	Appreciation of CHWs and home visit	“No, what I like about the programme is that you guys worked because it helped a lot of people, you see. It helped people who were in need and those who lacked knowledge.” “But we are very grateful of your help. I hope you’ll continue helping others, even though our months are coming to an end, but at-least if you continue helping others, you see.”
	Change of beliefs after visit/referral	“So now I saw the importance of exercising, it’s to keep myself healthy.” “Yes, because I have seen that when you take your treatment it reduces so the treatment is important.”
Factors influencing acting on the referral	Receipt of previous referral	“No, I’ve never received it.” “No, it was the first.”
	Barriers to utilizing referral	“I have 3 kids that I have to take care of.” “Walking is not safe you know?” “But it’s going to hinder me in the coming weeks because of the overcrowding there.”
	Facilitators to utilizing referral	“It’s not that far because it’s a walking distance from here.” “I enjoy the vibe there then it also gives me energy and motivates me.” “No, it was not hard it was easy.”
	What would make it easier to utilize referral	“I was happy with the process, like I don’t think there’s anything. The process was fine, nothing was a hassle.” “Nothing, I just need to get myself there.” “As I said that’s it’s the long lines in the clinic, going up and down, being sent from here to there.”
Perceived benefits of acting on the referral	Benefits of utilizing referral	“It helps me a lot, I don’t feel chest pains. No more headaches I’m a person who is always energetic.” “It’s so much fun. It reduces stress, you come back singing, you feel free when you come back home, you feel like going back there.” “I do see some difference when going to the clinic and they give me pills I think at least I get some changes and have some hope for the best.”
	Consequences of not utilizing referral	“When you don’t go to the clinic, you’re disadvantaging yourself.” “There are really no benefits of not doing something healthy.” “If I do go to the clinic, I think they can tell me what to do to reduce it.”

**Table 4:** Summary of main themes revealed through follow-up interviews

Main Themes	Sub-themes	Main ideas revealed through interviews
Prior knowledge and thoughts on BP	Knowledge about high BP	• Little knowledge about high BP • Results from poor diet and lack of PA
	Causes/symptoms	• Causes: poor diet, lack of PA, stress • Symptoms: dizziness, headaches, stroke, heart problems
	How to lower high BP	• Maintain healthier diet (*Females*), exercise regularly, Reduce stress
	Previous attempts to lower BP	• Majority - no previous attempts to lower BP • Few – improve diet or take medication
Psychosocial factors associated with BP control	Social norms of high BP	• Friends and family with high BP attempt to control by visiting health clinic and taking medication (*No Clinic*) • Friends and family with high BP attempt to control by engaging in lifestyle changes (*PA*)
	Seriousness of high BP	• Very serious and important to treat (*PA, Clinic*) • Not a serious health condition (*No Clinic*)
	Permission to utilize referral	• Do not require permission (i.e., from family members) to utilize referral
Perceptions of receiving the referral	Thoughts about receiving referral	• Happy, excited for new experience, helped them focus on health, while fewer were nervous about their health after receiving results (*PA*) • Scared, shocked, and stressed after learning about their elevated BP, but felt that the referral could help them (*No PA*)
	Appreciation of CHWs, home visit, and referral	• Appreciation for CHWs and home visits • Visit increased knowledge, raised awareness, and was encouraging
	Change of beliefs after visit/referral	• Increased PA levels • Importance of checking and treating high BP
Factors influencing acting on the referral	Receipt of previous referral	• First time receiving a referral to PA program or health clinic
	Barriers to utilizing referral	• Other responsibilities, lack of motivation, financial challenges, childcare issues, safety concerns (*No PA)* • Safety and childcare concerns (*Females, No PA*) • Site-specific barriers (i.e., long lines, far distance, overcrowding) (*No Clinic*)
	Facilitators to utilizing referral	• Within walking distance, PA program was motivating, process of using referral was easy (*PA*) • Health clinic was within walking distance (*Clinic)*
	What would make it easier to utilize referral	• Quicker service at health clinic (*Clinic*) • More internal motivation and financial assistance
Perceived benefits of acting on referral	Benefits of utilizing referral	• Less stressed, fewer symptoms, lower BP, enjoyed the program (*PA*) • Lower BP, starting medication (*Clinic*)
	Consequences of not utilizing referral	• Missed opportunity to receive help, BP will remain high, disadvantaging themselves (*No PA*)

PA: more reported by those who utilized their referral to the physical activity program; No PA: more reported by those who did not utilize their referral to the physical activity program; Clinic: more reported by those who utilized their referral to the clinic; No Clinic: more reported by those who did not utilize their referral to the clinic; Females: more reported by females; Males: more reported by males

#### Prior knowledge and thoughts on BP

Initially, many participants reported having limited knowledge about elevated or high BP (or ‘high blood’ as it is referred to locally), but as the interviews progressed, they demonstrated more knowledge about BP. Though many recognized that high BP resulted from a poor diet and a lack of PA, only a few participants reported knowing that high BP was a serious health condition. In addition to diet and exercise, participants felt that stress was also a key factor in causing high BP. Participants shared that some of the symptoms of high BP were dizziness, headaches, stroke, and heart problems. To lower ones BP, participants felt that they needed to maintain a healthier diet, which was mentioned more frequently by women, exercise regularly, and reduce their stress. A few participants also mentioned taking medication or reducing alcohol consumption as ways to lower their BP. When asked how they had tried to lower their BP in the past, most participants responded that they had not tried anything, while a few reported trying either to improve their diet or taking medication.

#### Psychosocial factors associated with BP control

In examining social norms, most participants knew at least one person with high BP, most commonly elderly individuals. These individuals attempted to control their high BP through visiting a health clinic and taking their medication. Overall, there were no differences in utilizing referrals between participants that did or did not know someone with high BP. Those who received a referral to a local health clinic, and did not go, more frequently reported knowing people with high BP seeking treatment at a health clinic and/or taking medication. Conversely, participants who received a referral, and went to the PA program, reported that people close to them with high BP were engaging in lifestyle changes, such as increasing their exercise and improving their diet. Responses varied when participants were asked how serious they believed high BP was to their health. Those who utilized their referral, either to the PA program or a local health clinic, reported feeling that high BP was very serious and important to check and treat. However, those who received a referral to a local health clinic and did not go, felt that high BP was not a serious health condition. In discussing their control over utilizing the referral, participants reported that they did not require permission (perceived control) from anyone (i.e., a family member) to attend either the PA program or a local health clinic.

#### Perceptions about receiving the referral

Overall, there was appreciation from participants across all groups for the CHWs, the work they do, and the home visit. The participants were thankful and felt that the CHWs were doing great work, and that the home visit was helpful, increased their knowledge, raised awareness of their health, and was encouraging. When asked about their thoughts and feelings when the CHWs first provided them with a referral, participants that went to the PA program reported having a positive reaction, such as feeling happy and excited for a new experience, and that the referral helped them focus on their health. A few of these individuals reported being nervous about their health after receiving their BP results. Those that did not attend the PA program reported being scared, shocked, and stressed to learn about their elevated BP, but also felt that the home visit and access to individualized referrals was a step in the right direction. Stress and fear about their health and their prognosis was prevalent among all that received a referral to a local health clinic. Participants in all groups reported that the home visit and receiving the referral changed their beliefs and actions, leading them to increase their PA levels and understanding the importance of checking and treating their high BP.

#### Contextual factors influencing utilization of the referrals

Participants reported that this was the first time they had received a referral to a PA program or to a health clinic. Few barriers were reported by those who went to the PA program or a health clinic; however, financial considerations, childcare, and safety were still of concern for these individuals. Those who went to the PA program suggested that factors that facilitated their decision included that the gym was within walking distance from their home, the program was motivating, and the process of utilizing the referral was easy. Those who reported going to a health clinic also mentioned the walkable distance as a facilitator. Participants who did not go to the PA program reported multiple barriers to utilizing their referral, including having other responsibilities, lack of motivation, financial challenges, childcare issues, and safety concerns. Males who did not attend the PA program reported specific barriers, such as issues with their personal identification, lack of proper exercise attire, and losing the referral, whereas females were more concerned with their safety and childcare while they were gone. Participants who did not go to a clinic reported more site-specific barriers, such as having to travel a far distance, overcrowding at the clinic, and long wait times. When asked what would make it easier for them to utilize their referrals in the future, participants who received referrals to a local health clinic expressed wanting quicker service. Other notable responses across all groups included needing more internal motivation and financial assistance.

#### Perceived benefits of utilizing the referral

Those that attended the PA program reported multiple benefits including feeling better, being less stressed, having fewer health symptoms, lowering their BP, and enjoying the program. Those that went to a health clinic reported lowering their BP after starting medication. Those that did not utilize their referral, either to the PA program or a health clinic, reported feeling like they lost out on an opportunity to receive help and acknowledged that their BP would likely remain high. These participants admitted that they disadvantaged themselves by not utilizing their referral and several individuals that were referred to a health clinic acknowledge that they could have benefitted from learning how to lower their BP.

## Discussion

The need for new strategies to address NCDs in LMICs is critical. One potential solution is having CHWs conduct home visits to screen, identify, and connect at-risk patients to appropriate health resources in their local community. While CHWs have successfully performed home visits to treat diseases, such as HIV, tuberculosis and malaria [29–31], few studies have assessed the potential role of CHWs as connectors to local health resources for NCD prevention and treatment. This study assessed the proportion of community members that utilized referrals provided by CHWs during home visits and explored individual behavioral and contextual factors that influenced their decisions to utilize the referrals. Key findings from this study include higher than expected utilization of referrals to the local PA program and the identification of several enabling factors aiding and multiple barriers preventing participants from utilizing their referrals.

A high proportion (95.5%) of community members consented to the home-based screening, showing the potential for CHW-led home visits to increase healthcare access. Home visits are acceptable to community members and seen as convenient (as opposed to having to go elsewhere to get care), provide high quality care, and successfully increase access to care and health resources [21, 32]. Our approach resulted in the screening of a relatively large number of community members in a short period of time, identifying at-risk participants based on their BP levels, and increasing levels of health awareness in this under-resourced community. Providing referrals to participants during home visits also increased access to care, aligning with findings from a systematic review performed by Woldie et al. [33] who found that CHWs significantly improved utilization of health resources through increased access to medication and raising awareness about their health conditions. Future efforts building off our findings is to increase integration of the referral process with the local primary healthcare system to optimize CHW community outreach to screen and connect community members back to the health clinics for treatment and resources, further increasing penetration and healthcare access in low resource communities.

Even though many participants were initially shocked to learn that they had elevated BP or hypertension, there was an overall feeling of gratefulness and appreciativeness for the home visit and the referrals to health resources. Previous literature discusses the positive interactions and high levels of appreciation community members have with CHWs due to their rapport building [21, 34], which increases comfort levels when receiving health information or resources from members of their own community. Despite studies suggesting community member apprehension with CHW knowledge and expertise, [35] community members’ trust of CHWs has also been well documented in previous literature [36–38]. Previous work has demonstrated the potential effectiveness of CHWs connecting patients with type 2 diabetes mellitus to resources and increasing access to care in LMICs [39]. Participants revealed that this was the first referral they had received connecting them to health resources in the community, demonstrating a clear need for the development of better linkages to care in under-resourced communities.

The proportion of referral utilization (approximately one in four referred participants enrolled in the PA program) in our study is similar to previous work in individuals with elevated BP [40, 41] and demonstrates community member interest in engaging in health promoting activities when given the opportunity. The referral utilization in this study is relatively high compared to a study that looked at referral utilization to weight management program in which only 15.6% of participants engaged with the program [42]. The utilization of referrals to the PA program was a result of several enabling factors, including the close walking distance to the facility and ease of registering for the program. This aligns with previous work in LMICs, which demonstrated that resources offered closer to home were effective in reducing travel distance and cost barriers, resulting in increased access to healthcare resources [43]. Similarly, other studies have found that farther distances from health facilities was associated with lower utilization and reduced maternal healthcare [44, 45]. We also found greater referral utilization among those with higher levels of education, suggesting that more education may increase understanding of the health risks associated with elevated BP and the importance of engaging in healthy lifestyle choices. Other facilitators included being connected to a health resource that was enjoyable, improving their health, and perceiving their elevated BP to be a serious health condition. Previous studies report conflicting results regarding the influence that perception of health severity has on health resource utilization. One study found that maternal health resource utilization was not influenced by perceived need [46], while another study found that perceived severity and fear increased COVID-19 testing and health resource utilization [47]. Though this study found several facilitators to engagement in the PA program, it should be noted that changing the type of evidence-based intervention could theoretically change engagement levels.

Common across all participants when deciding to utilize their referrals was a need for childcare and a concern for their safety. Providing childcare for those who are seeking health resources is an important strategy; this is a particularly substantial barrier to PA particularly among women [48, 49] and can be exceptionally hindering for low-income women [50]. Participants that did not utilize their referral to the gym reported several barriers including having other responsibilities, a lack of motivation, financial challenges, childcare issues, and safety concerns. These findings are similar to those reported by Garmendia et al. [51] in assessing adherence to a PA intervention in Chile, a middle-income country, where similar barriers to PA engagement included poverty, high crime rates within the neighborhood, and other family and child responsibilities. Odunitan-Wayas et al. [52] also found that personal safety concerns hindered PA levels among those living in a low-income community in South Africa. The proportion of community members utilizing the referral to the PA program in the current study may reflect our offering a safe, protected environment for being physically active. Other unique barriers reported by males in our study deterring them from using their referral included losing the paper referral and a lack of proper exercise clothing. These findings suggest the need for additional follow up with participants, providing ongoing encouragement, and engaging in problem-solving. Portacio et al. [53] found that even a routine follow up with participants after a referral increased enrollment to a text messaging program for healthy behaviors from 3.9 to 42.3%. With poverty and unemployment rates so high in South Africa [54], providing financial incentive may also be another solution to improving referral utilization. A study conducted in Kenya and Uganda found that a one-time financial incentive improved patient connection to hypertension care by 30% [55].

The participants who did not utilize the referral to a local health clinic stated mostly site-specific barriers, including long wait times, overcrowding, and too far of a walk. Similar barriers have been reported in previous literature [21, 56, 57] Hasumi and Jacobsen [56] found that long wait times were the most commonly reported health resource problem in South Africa. Gulliford et al. [57] also discussed that long wait times can be an organizational barrier to access to care. Further, our participants reported not being influenced by knowing family and friends undergoing treatment for high BP and visiting a health clinic. Though vicarious experiences have been found to have positive effects on utilization of different health resources in high income countries [58–60], it is possible that in the context of this under-resourced community in South Africa participants may have been discouraged observing the difficulties their friends and family faced in seeking assistance for their high BP at local health clinics (e.g., long lines, medication side effects, high costs). This is supported by previous work that found that medication side effects and finances were major barriers to treatment adherence for hypertension in South Africa [61]. Additionally, these individuals did not feel that high BP was a serious issue, had not experienced severe symptoms, and/or had limited knowledge about high BP. This aligns with previous work demonstrating that those who do not experience symptoms of high BP are less likely to seek treatment [62]. Previous literature that assessed referral utilization to health resources for communicable diseases, such as tuberculosis and HIV, found higher rates of linkage to health resources which may be due to the fact that these diseases have more pronounced symptoms compared to elevated BP [63]. Additionally, it is not uncommon for individuals to see family and friends sent home from health clinics with no treatment plan for their high BP. Treatment is usually held back unless BP is exceptionally high in resource-limited, primary care due to guidelines only suggesting to treat patients with BP > 160/>100 with medication [11, 12].

Several limitations were present in this study. First, there was an unexpectedly large number of participants who refused to accept the referral from the CHWs potentially limiting the generalizability of our work to other communities. Anecdotally, some participants mentioned already having a future appointment at a health clinic. We also did not control the selection or size of community where the CHWs conducted the home screenings as this was a predetermined part of their training, which may have contributed to selection bias. Further, workflow issues with the CHWs administering the referrals lead to some miscommunication of who should receive which type of referral. However, we were still able to track and follow up with all community members referred to the PA program, partially negating this limitation. Second, we were unable to track participants who may have utilized their referral at any number of local health clinics across Soweto. Our inability to conduct follow-up interviews with an equal number of participants referred to the health clinics may be due to participants not wanting to share that they did not utilize the referral or changes in their contact information. Mobile phone theft and crime rates are rife in Soweto causing a constant changing of contact information [64]. Future work should explore the acceptance and utilization of referrals to the local health clinics. Third, we were unsuccessful in completing interviews with an equal number of participants from each of the four groups, particularly those receiving referrals to a local health clinic, which partially limits our ability to generalize information about the decision-making processes of these individuals.

This study also has several strengths. The design of this study was a pragmatic, real-world trial that did not require invasive intervention or follow up. Though we were unable to control for confounding factors, pragmatic designs have multiple advantages including increasing overall generalizability and evaluating the real-world application of interventions and programs [65]. Additionally, our work reached a large number of community members over a short period of time and the home visits were very well received by the local community, confirming our previous work demonstrating the acceptability of the home visits [21]. Finally, the mixed methods design of this study combines the strengths of both quantitative and qualitative design and addresses the weaknesses of each type of design [66]. Through the quantitative component of the study, we identified the acceptance and utilization of referrals to the PA program. The qualitative component, using purposeful sampling, provided us with a greater understanding of the reasons behind participant decisions to utilize their referral [67].

## Conclusion

This study was successful in demonstrating that using CHWs to conduct home visits and provide individualized referrals based on BP screenings has the potential to connect many individuals to appropriate health resources to manage their elevated BP. Further, a good proportion of community members utilized their referrals and joined a local community-based PA program, demonstrating a willingness to engage in health promoting activities when provided the opportunity. Greater attention is needed to address and overcome commonly reported barriers and community member hesitancy to utilize health resources. Overall, this study shows the potential of CHWs to identify and address the growing burden of uncontrolled hypertension, and potentially other NCDs, by increasing health screenings and access to health resources through semi-customized referrals in under-resourced communities.

### Electronic supplementary material

Below is the link to the electronic supplementary material.

**Additional File 1:** Semi-structured interview guides mapped onto Andersen’s Expanded Behavioral Model

## Data Availability

The datasets used and analyzed during the current study are available from the corresponding author on reasonable request.

## Abbreviations

BP: blood pressure
CHWs: community health workers
DBP: diastolic blood pressure
LMICs: low- and middle-income countries
NCDs: non-communicable diseases
PA: physical activity
SBP: systolic blood pressure
