# Supplementary material for: Examining the referral of patients with elevated blood pressure to health resources in an under-resourced community in South Africa
Source: BMC Public Health. 2024 Feb 8;24:412. doi: 10.1186/s12889-023-17359-z (PMC10854044; doi:10.1186/s12889-023-17359-z)
Supplement: Supplementary file 1 — Additional File 1: Semi-structured interview guides mapped onto Andersen’s Expanded Behavioral Model [file 12889_2023_17359_MOESM1_ESM.docx]

**Additional file 1. Interview guide based on Andersen’s Expanded Behavioural Model of Health Service Use.**

**Interview Script for Individuals Referred to Exercise Programming (Pre-Hypertension)**

This interview guide is for used for individuals with a blood pressure of 120-139/80 and who received a referral to the exercise programme.

Demographic Information

Can you tell us your age?

Are you currently: Working Retired Unemployed Other

Do you have medical insurance? Yes No

| **Andersen’s Expanded Behavioural Model** | |
| --- | --- |
| **Psychosocial Factors** |  |
| Knowledge | - What do you know about blood pressure or ‘high blood’? |
| Social Norms | - How many people of your age group do you know with high blood? - What do you think these people usually do about their high blood? |
| Perceived Control | - How big a problem is this high blood pressure for you? - How important is it to you to get help for your blood pressure at the moment? - How much do you think you can lower your blood pressure on your own (without help)? - Would you need help or permission from anyone else to go to an exercise programme? |
| Attitudes | - When our health advocate visited your home, did you receive a referral? - What referral did you receive? What did you think about this? - Why do you think you were given this referral? - Have you received a referral like this from anyone in the past? - How did you feel about receiving this referral? - Did you decide then if you would go or not go? Why was that? |
| **Enabling Factors** |  |
| Previous referral *(skip questions if no previous referral)* | - Please tell us about the last time you had a referral for exercise. What happened? - Did you go? - What was the outcome if you went? OR - If you didn’t go, what made you decide not to go? |
| Current Referral | - About the referral you received from our team, how far away is the exercise programme from where you live? - How easy is it for you to travel there? |
| Current Referral *(ask only if they attended)* | - How easy was it for you to go to the exercise programme? - What were some things that helped you attend the exercise programme? - What would make it even easier for you to attend the programme in the future? |
| Current Referral *(ask if they did not attend)* | - What stopped you from going to the exercise programme? - What would it take for you to go the exercise programme? - What else might help you attend the exercise programme in the future? |
| **Need** |  |
|  | - How serious do you think your high blood pressure is? - Did you know or think that your blood pressure was a little high before our team measured it? Why was this? - Have you done anything before to lower your blood pressure? - Right now, how much do you want to lower your blood pressure? |
| Current Referral *(ask only if they attended)* | - What do think will happen to your blood pressure because you went to the exercise programme? - What are the benefits to you of going? |
| Current Referral *(ask if they did not attend)* | - What do think will happen to your blood pressure because you did not go to the exercise programme? - What are the benefits to you of not going? |
| **Other** | - Is there anything else you would like to share with us about this? |

**Interview Script for Individuals Referred to Primary Healthcare Clinic (Hypertension)**

This interview guide is for used for individuals with a blood pressure ≥140/90 that received a referral to their primary healthcare centre.

Demographic Information

Can you tell us your age?

Are you currently: Working Retired Unemployed Other

Do you have medical insurance? Yes No

| **Andersen’s Expanded Behavioural Model** | |
| --- | --- |
| **Psychosocial Factors** |  |
| Knowledge | - What do you know about blood pressure or ‘high blood’? |
| Social Norms | - How many people of your age group do you know with high blood? - What do you think these people usually do about their high blood? |
| Perceived Control | - How big a problem is this high blood pressure for you? - How important is it to you to get help for your blood pressure at the moment? - How much do you think you can lower your blood pressure on your own (without help)? - Would you need help or permission from anyone else to go a health clinic? |
| Attitudes | - When our health advocate visited your home, did you receive a referral? - What referral did you receive? What did you think about this? - Why do you think you were given this referral? - Have you received a referral like this from anyone in the past? - How did you feel about receiving this referral? |
| **Enabling Factors** |  |
| Previous referral to a health clinic *(skip questions if no previous referral)* | - Please tell us about the last time you had a referral to a health clinic. What happened? - Did you go? - What was the outcome if you went? OR - If you didn’t go, what made you decide not to go? |
| Current Referral | - About the referral you received from our team, how far away is the health clinic from where you live? - How easy is it for you to travel there? |
| Current Referral *(ask only if they attended)* | - How easy was it for you to go to the health clinic? - What were some things that helped you go to the health clinic? - What would make it even easier for you to go to the health clinic in the future? |
| Current Referral *(ask if they did not attend)* | - What stopped you from going to the health clinic? - What would it take for you to go the health clinic? - What else would help you go to the health clinic in the future? |
| **Need** |  |
|  | - How serious do you think your high blood pressure is? - Did you know or think that your blood pressure was high before our team measured it? Why was this? - Have you done anything before to lower your blood pressure? - Right now, how much do you want to lower your blood pressure? |
| Current Referral *(ask only if they attended)* | - What do think will happen because you went to the health clinic? - What are the benefits to you of going? |
| Current Referral *(ask if they did not attend)* | - What do think will happen because you did not go to the health clinic? - What are the benefits to you of not going? |
| **Other** | - Is there anything else you would like to share with us about this? |
